# SREBF1-based metabolic reprogramming in prostate cancer promotes tumor ferroptosis resistance

**DOI:** 10.1038/s41420-025-02354-7

**Published:** 2025-02-23

**Authors:** Guojiang Wei, Ying Huang, Wenya Li, Yuxin Xie, Deyi Zhang, Yuanjie Niu, Yang Zhao

**Affiliations:** 1 Department of Radiology, The Second Hospital of Tianjin Medical University, Tianjin, People’s Republic of China; 2 Tianjin Institute of Urology, The Second Hospital of Tianjin Medical University, Tianjin, People’s Republic of China; 3 Department of Urology, The Second Hospital of Tianjin Medical University, Tianjin, People’s Republic of China; 4 Department of Urology, Tianjin Medical University General Hospital, Tianjin, People’s Republic of China

**Keywords:** Prostate cancer, Fatty acids, Next-generation sequencing, Gene regulation

## Abstract

Metabolic reprogramming in prostate cancer has been widely recognized as a promoter of tumor progression and treatment resistance. This study investigated its association with ferroptosis resistance in prostate cancer and explored its therapeutic potential. In this study, we identified differences in the epithelial characteristics between normal prostate tissue and tissues of various types of prostate cancer using single-cell sequencing. Through transcription factor regulatory network analysis, we focused on the candidate transcription factor, SREBF1. We identified the differences in SREBF1 transcriptional activity and its association with ferroptosis, and further verified this association using hdWGCNA. We constructed a risk score based on SREBF1 target genes associated with the biochemical recurrence of prostate cancer by combining bulk RNA analysis. Finally, we verified the effects of the SREBPs inhibitor Betulin on the treatment of prostate cancer and its chemosensitization effect. We observed characteristic differences in fatty acid and cholesterol metabolism between normal prostate tissue and prostate cancer tissue, identifying high transcriptional activity of SREBF1 in prostate cancer tissue. This indicates that SREBF1 is crucial for the metabolic reprogramming of prostate cancer, and that its mediated metabolic changes promoted ferroptosis resistance in prostate cancer in multiple ways. SREBF1 target genes are associated with biochemical recurrence of prostate cancer. Finally, our experiments verified that SREBF1 inhibitors can significantly promote an increase in ROS, the decrease in GSH, and the decrease in mitochondrial membrane potential in prostate cancer cells and confirmed their chemosensitization effect in vivo. Our findings highlighted a close association between SREBF1 and ferroptosis resistance in prostate cancer. SREBF1 significantly influences metabolic reprogramming in prostate cancer cells, leading to ferroptosis resistance. Importantly, our results demonstrated that SREBF1 inhibitors can significantly enhance the therapeutic effect and chemosensitization of prostate cancer, suggesting a promising therapeutic potential for the treatment of prostate cancer.

## Introduction

Prostate cancer is the most prevalent malignant tumor among men in Europe and the United States, ranking as the second leading cause of male cancer-related deaths [1]. This disease is highly heterogeneous, with its progression linked to multiple gene deletions or mutations, including FOXA1, ZNF292, CHD1, PTEN, and TP53 [2, 3]. Prostate growth and development are dependent on androgens, and androgen deprivation therapy (ADT) remains the cornerstone of current prostate cancer treatment [4]. However, due to the complex heterogeneity of prostate cancer, the therapeutic efficacy of ADT can vary significantly, thereby affecting patient prognosis. Furthermore, nearly all patients eventually develop resistance to castration therapy after a certain treatment period [5]. The mechanisms underlying castration resistance are extremely complex and ultimately result in the failure of treatments targeting the androgen receptor (AR) signaling pathway. Therefore, there is an urgent need to identify novel therapeutic targets for prostate cancer.

Metabolic reprogramming is a distinct characteristic of prostate cancer. Normal prostate tissue exhibits unique metabolic features, notably the secretion of large amounts of citric acid, a semen component that is abundantly synthesized in prostate cells [6, 7]. Citric acid serves as a crucial hub linking cell metabolism, particularly by mediating lipid metabolism through the amphibolic pathway. Consequently, the substantial secretion of citric acid from normal prostate tissues can be utilized to synthesize fatty acids and cholesterol during cancer development [8]. On one hand, the metabolic shift in prostate cancer enables cancer cells to adapt to their energy requirements, while simultaneously, the utilization of cholesterol for the synthesis of endogenous steroid hormones promotes the proliferation of prostate cancer cells [9]. Consequently, exploring the metabolic vulnerabilities in prostate cancer may provide a direction for novel treatment strategies.

Ferroptosis is a unique form of iron-dependent cell death characterized by the accumulation of excessive lipid peroxidation in the cell membrane [10]. Resistance to ferroptosis not only facilitates tumor development but also contributes to tumor resistance to treatment [11]. Multiple mechanisms are involved in ferroptosis resistance in prostate cancer. Liang et al. first reported that AR induce ferroptosis resistance in prostate cancer by regulating the activity of MBOAT2 [12]. Yi et al. demonstrated that SREBPs activation mediated by the PI3K-AKT-mTOR pathway can promote ferroptosis resistance in prostate cancer [13]. However, the relationship between SREBPs-mediated metabolic reprogramming and ferroptosis resistance in prostate cancer remains unclear.

We confirmed the association between SREBF1-mediated metabolic reprogramming in prostate cancer and ferroptosis in human samples, using a combination of single-cell sequencing and Bulk-RNA analysis. Additionally, we validated SREBF1 as a potential therapeutic vulnerability and an effective target for prostate cancer by employing SREBF1 inhibitors.

## Results

### Overview of single-cell sequencing characteristics of normal prostate tissue and different types of prostate cancers

**Fig. 1:**
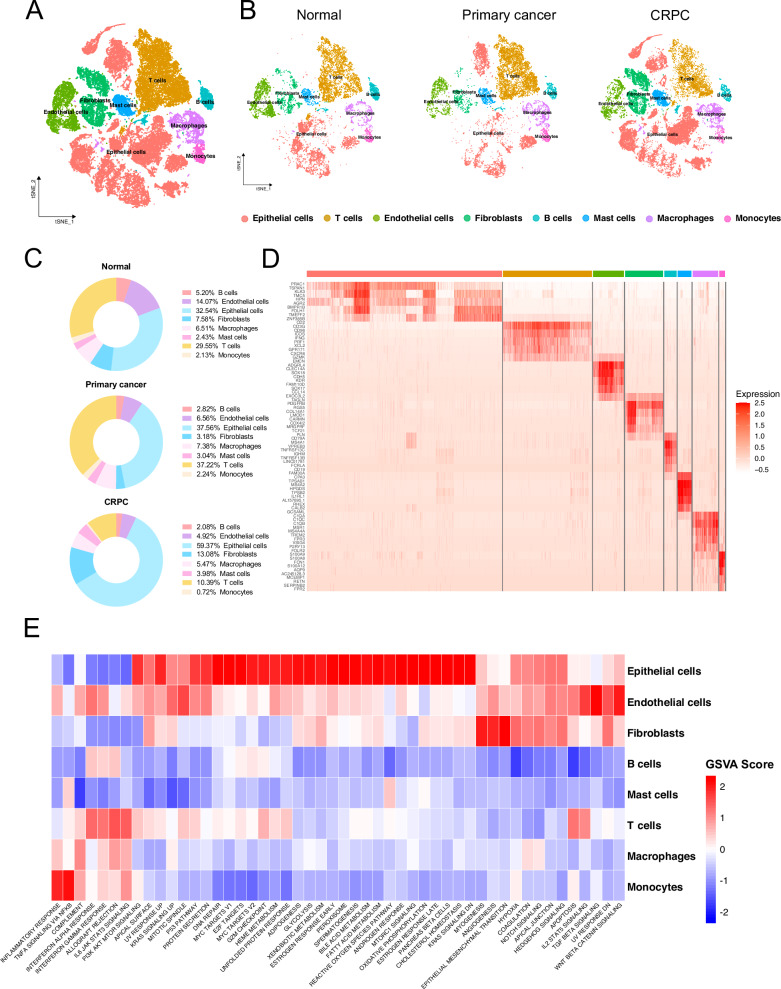
Overview of single-cell RNA sequencing characteristics of prostate cancer. **A** tSNE plot showing cell clusters after dimensionality reduction and cell type annotation following quality control. **B** tSNE plot displaying facet diagrams from Normal, Primary cancer, and CRPC samples. **C** Distribution of different cell types in Normal, primary cancer, and CRPC samples. **D** Heatmap of the top 10 differentially expressed genes for each cell type. **E** Heatmap of Gene Set Variation Analysis (GSVA) based on the average gene expression for each cell type. Gene set: Hallmark from MSigDB.

Our study included normal prostate tissue cells, prostate cancer cells from radical prostatectomy (RP) samples representing primary cancer, and prostate cancer cells from castration-resistant prostate cancer (CRPC) samples. Following quality control (QC), a total of 51,092 cells were included in the study. Subsequently, all cells were divided into 33 clusters by setting the resolution to 0.6. These clusters were annotated based on characteristic gene expression differences, identifying T cells, B cells, Macrophages, Endothelial cells, Fibroblasts, Mast cells, Monocytes, and Epithelial cells (Fig. 1A). It is noteworthy that regardless of the sample origin, epithelial cells constituted the predominant cell type, accounting for 32.54%, 37.56%, and 59.37% of normal, primary cancer, and CRPC samples, respectively. Interestingly, in CRPC-derived samples, the proportion of T cells significantly decreased, accounting for only 10.39% of all cells, compared to 29.55% and 37.22% in normal and primary cancer samples, respectively (Fig. 1B, C). Differential gene expression analysis identified several highly expressed genes in epithelial cells, including KLK3, PRAC1, and TSPAN1. KLK3, which encodes the prostate-specific antigen (PSA), is specifically expressed in prostate tissues, and is widely used in prostate cancer screening. Prostate Cancer Susceptibility Candidate Protein 1(PRAC1) is highly expressed in the prostate [14]. TSPAN1 is upregulated in various cancers and is regulated by androgens, promoting the proliferation and migration of prostate cancer [15] (Fig. 1D). Furthermore, we identified characteristic differences between different clusters through Gene Set Variation Analysis (GSVA) of the average expression levels. Epithelial cells exhibited higher activity in pathways, such as the PI3K-AKT pathway, P53 pathway, MYC, androgen response, glycolysis, fatty acid metabolism, and bile acid metabolism. T cells showed high activity in the interferon, IL-2, and IL-6 signaling pathways, whereas fibroblasts exhibited uniquely high activity in the epithelial-mesenchymal transition pathway (Fig. 1E).

### Characteristics of different types of epithelial cells

**Fig. 2:**
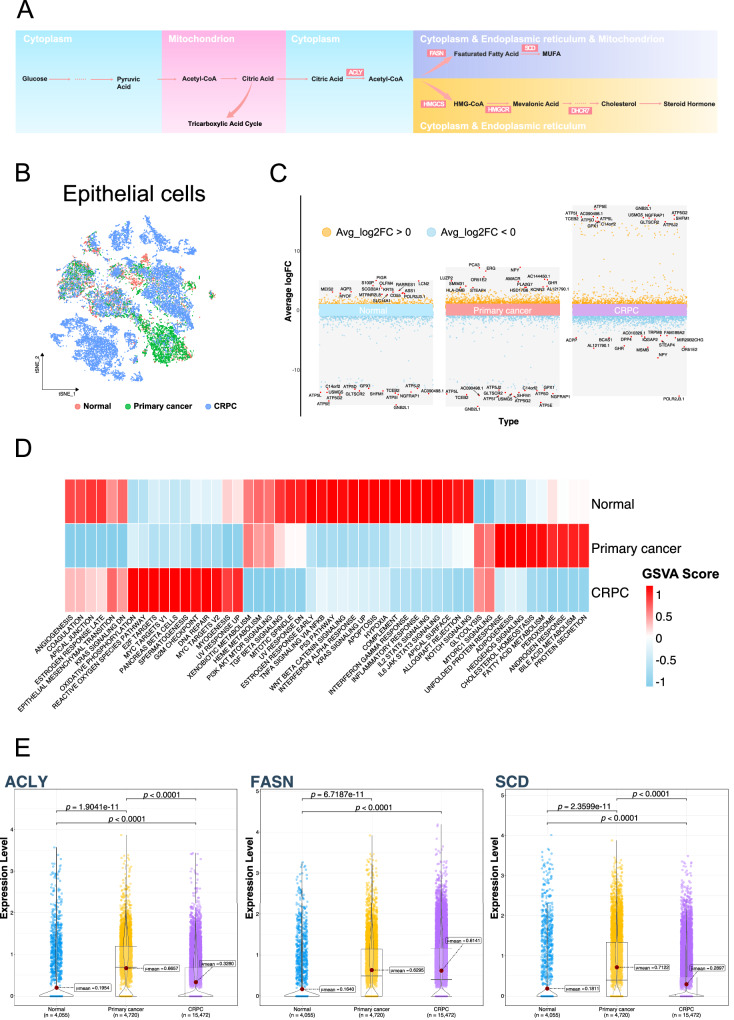
Characteristic differences in epithelial cells of prostate tissue and different types of prostate cancer. **A** Schematic diagram of intracellular energy metabolism, with highlighted products and enzymes. **B** tSNE plot of epithelial cells, colored by sample types: Normal, Primary cancer, and CRPC. **C** Differential gene analysis in epithelial cells from Normal, Primary cancer, and CRPC samples, showing genes with the largest average log fold change (LogFC). **D** Heatmap displaying the average gene set variation analysis (GSVA) scores in epithelial cells from Normal, Primary cancer, and CRPC samples. The gene set used is Hallmark from MSigDB. **E** Violin plot showing the expression levels of ACLY, FASN, and SCD genes in epithelial cells from Normal, Primary cancer, and CRPC samples.

Given the lipid-related metabolic signatures observed in epithelial cells, we further analyzed the epithelial cells (Fig. 2B). Citrate, as a hub linking glycolysis and lipid metabolism, including fatty acid and cholesterol synthesis, plays an important role in prostate cancer (Fig. 2A). Through gene differential analysis, we identified significant gene expression differences in epithelial cells from normal, primary cancer, and CRPC samples. In the primary cancer samples, the expression of PCA3, ERG, NPY, and AMACR was significantly upregulated, making them the characteristic genes with the largest expression differences. PCA3, a long non-coding RNA (lncRNA), is highly expressed specifically in prostate cancer and has been used for urine detection of prostate cancer [16]. The ERG gene serves as a prostate cancer marker, and the ERG-TMPRSS gene fusion is one of the most common gene rearrangements in prostate cancer [17]. AMACR holds significant value in the pathological diagnosis of prostate cancer, serving as a characteristic marker, and its expression is closely related to the fatty acid metabolism in prostate cancer [18]. ATP-related genes were significantly upregulated in the CRPC cells (Fig. 2C). Subsequently, we observed significant differences in the characteristics of the epithelial cells derived from these three sources using GSVA. In normal samples, some inflammation-related pathways showed higher activity, whereas epithelial cells derived from primary cancer samples mainly exhibited higher activity in androgen response, fatty acid metabolism, cholesterol metabolism, and other pathways. Epithelial cells derived from the CRPC samples were primarily concentrated in the E2F, MYC, and DNA repair pathways. Epithelial cells derived from primary cancer and CRPC samples showed higher activity in the glycolysis and MTORC1 pathways (Fig. 2D). Overexpression of ACLY, a key gene in the flow of citric acid to lipid metabolism, was observed in epithelial cells derived from tumor samples. The average expression levels in primary cancer samples were 0.6657, while in CRPC samples, it was 0.3280, and in normal samples, it was only 0.1954. FASN, a key gene in mediating fatty acid metabolism, also exhibited high expression levels in primary cancer samples and CRPC samples, with values of 0.6295 and 0.6141, respectively, compared to 0.1640 in normal samples. SCD, which mediates the formation of monounsaturated fatty acids (MUFA) and is related to ferroptosis resistance, showed expression levels of 0.7122, 0.2897, and 0.1811 in primary cancer, CRPC, and normal samples, respectively (Fig. 2E).

### Transcription factor regulatory network of prostate epithelial cells

**Fig. 3:**
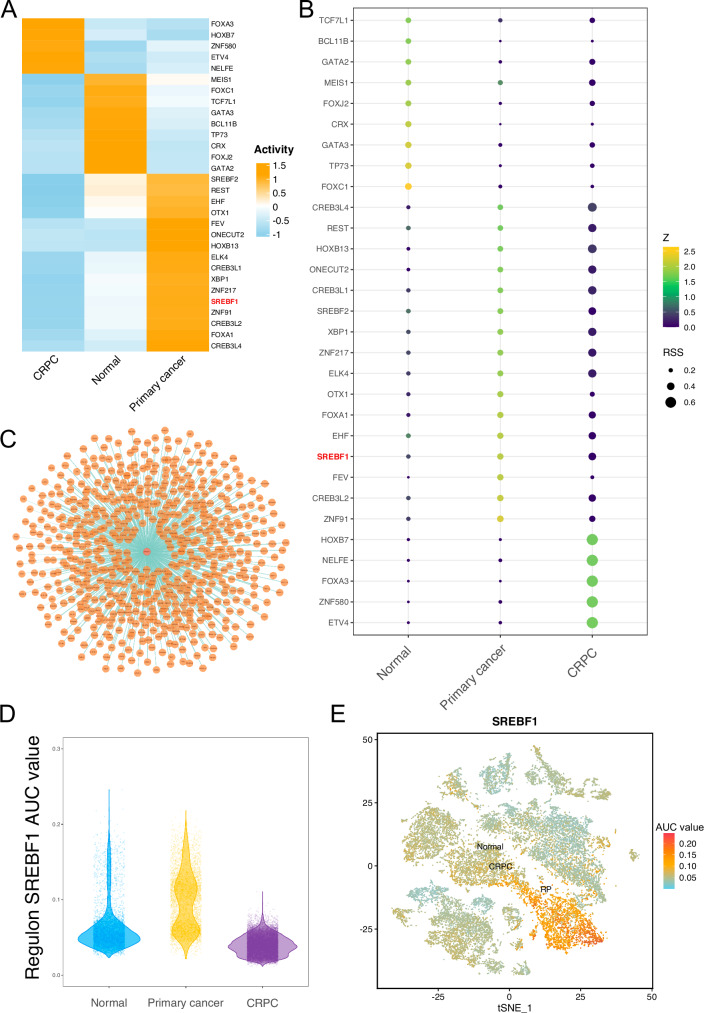
Analysis of transcription factors in prostate cancer epithelial cells. **A** Heatmap showing the average Area Under the Curve (AUC) values of 30 transcription factors. **B** RSS plot of 30 transcription factors. Color represents the Z value, and the size of the circle represents the RSS value. **C** Visualization of SREBF1 and its target genes. **D** Violin plot showing the AUC values of SREBF1 in epithelial cells from Normal, Primary cancer, and CRPC samples. **E** tSNE plot displaying the expression of SREBF1 in epithelial cells from Normal, Primary cancer, and CRPC samples. Color represents the AUC value.

We analyzed the transcription factor activity in epithelial cells using SCENIC, resulting in the identification of 219 transcription factors. Among these, 30 transcription factors exhibited a higher activity based on an RSS value greater than 0.2 and a Z value greater than 1.4. These included SREBF1, SREBF2, and FOXA1 (Table S1). The average area under the curve (AUC) of the regulons in each group was calculated. The activities of SREBF1 and FOXA1 were higher in the primary cancer samples, while the activities of FOXC1 and TP73 were higher in normal samples, and the activities of transcription factors such as FOXA3 were higher in the CRPC samples (Fig. 3A). The RSS values of SREBF1 were higher in primary cancer and CRPC samples, with values of 0.32 and 0.40, respectively, while the value was only 0.21 in the Normal sample (Fig. 3B). We visualized the target genes of SREBF1, including HMGCS1, DHCR7, SC5D, SCD1, ACLY, FASN, and LDLR. (Fig. 3C and Table S2). Furthermore, the transcriptional activity of SREBF1 was higher in primary cancer samples (Fig. 3D, E).

### Characteristic differences based on SREBF1 transcriptional activity grouping

**Fig. 4:**
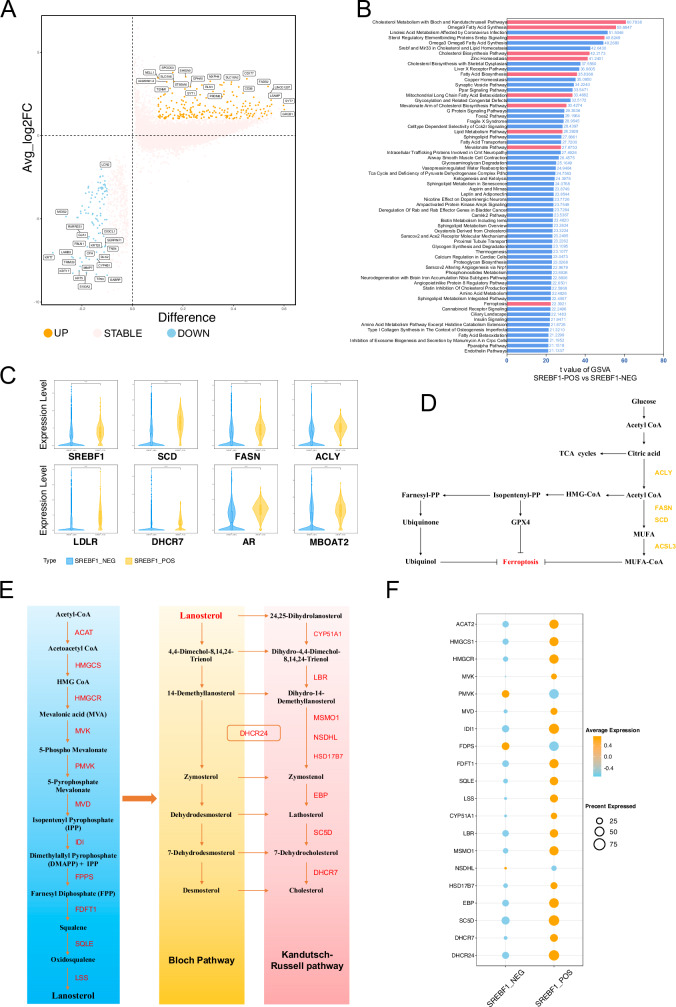
Differences between the two groups SREBF1_POS and SREBF1_NEG. **A** Volcano plot showing the differential gene expression analysis between SREBF1_POS and SREBF1_NEG groups. **B** Gene Set Variation Analysis (GSVA) comparing the SREBF1_POS and SREBF1_NEG groups. The gene set used is CP:WIKIPATHWAYS from MSigDB. **C** Violin plot displaying the expression levels of selected genes between SREBF1_POS and SREBF1_NEG groups. **D** Pathway map of ferroptosis and lipid metabolism. **E** Schematic diagram of cholesterol synthesis, from acetyl-CoA to cholesterol. **F** Dot plot showing the expression levels of cholesterol synthesis-related genes between SREBF1_POS and SREBF1_NEG groups.

We divided all cells into SREBF1-positive and SREBF1-negative groups based on the binary regulon AUC matrix in SCENIC, and analyzed the differences between the two groups (Fig. 4A). Notably, “positive” and “negative” mentioned above do not represent the presence or absence of SREBF1 activity, but only the classification of the binary results of regulon AUC, representing the level of SREBF1 activity in cells. We conducted GSVA on the two groups and found significant differences in the pathways related to cholesterol and fatty acid metabolism. Among them, the “Cholesterol Metabolism with Bloch and Kandutsch-Russell Pathways” showed the most obvious enrichment difference (t Value: 60.78), while in fatty acid metabolism, “Omega9 Fatty Acid Synthesis” ranked second (t Value: 55.68). Both the Bloch and Kandutsch-Russell pathways are involved in cholesterol synthesis. Omega 9 Fatty Acid (MUFA) Synthesis is associated with anti-ferroptosis and is mainly regulated by SCD. Other pathways, such as the “Mevalonate Arm of Cholesterol Biosynthesis Pathway” (t value: 30.43) and “Mevalonate Pathway” (t Value: 27.68), also showed significant differences. Cholesterol can synthesize endogenous androgens to promote prostate cancer cell proliferation, and high activity of the Mevalonate (MVA) pathway is associated with resistance to ferroptosis (Fig. 4B, D). We compared differences in the expression of some SREBF1 targeted genes related to ferroptosis between the two groups. SCD, FASN, and ACLY were significantly upregulated in SREBF1-positive cells, while cholesterol-related LDLR and DHCR7 were also significantly up-regulated in SREBF1-positive cells. Additionally, we observed the up-regulation of AR and MBOAT2, which are involved in the anti-ferroptosis mechanism of MBOAT2, in SREBF1-positive cells (Fig. 4C).

Furthermore, we focused on the gene expression differences in the cholesterol synthesis pathway, from acetyl-CoA to cholesterol (Fig. 4E). Almost all genes were highly expressed in the SREBF1-positive group, with a higher expression ratio. Only PMVK and FDPS showed higher average expression levels in the SREBF1-negative cells, but their expression percentages were lower. This indicated that the cholesterol synthesis pathway was highly activated in the SREBF1-positive group, with an average expression percentage of genes in 7-dehydrocholesterol (7-DHC) synthesis, including EBP (81.05%), SC5D (95.72%), and MSMO1 (73.30%), which was significantly higher than that of DHCR7 (54.94%) (Fig. 4F).

### Single-cell sequencing hdWGCNA identifies modules associated with SREBF1 and their characteristics

**Fig. 5:**
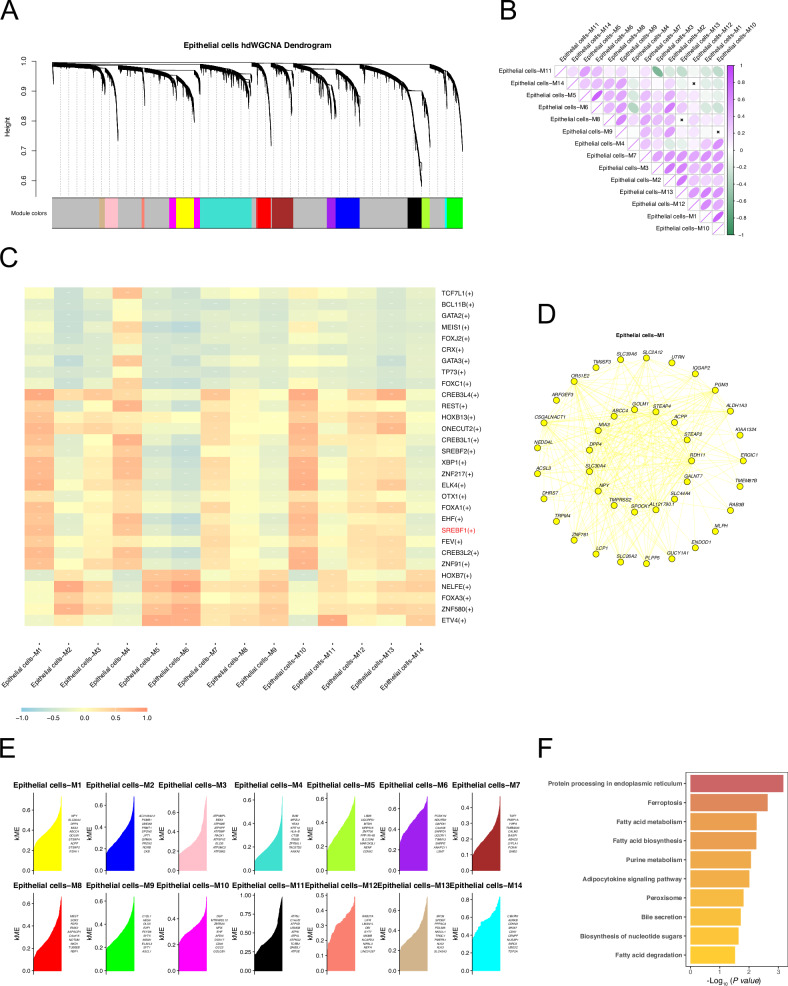
The relationship between SREBF1 and ferroptosis revealed by hdWGCNA. **A** Dendrogram of hdWGCNA in prostate cancer epithelial cells. **B** Correlation diagram between modules identified by hdWGCNA. **C** Correlation analysis between the modules identified by hdWGCNA and the transcription factor activities identified by SCENIC. **D** Top 40 hub genes of Epithelial cells-M1 module. **E** Hubgenes of all modules identified by hdWGCNA, ranked by kME. **F** KEGG pathway enrichment analysis of hub genes in the Epithelial cells-M1 module.

We performed hdWGCNA analysis of epithelial cell subsets. First, the topological overlap matrix (TOM) was calculated by selecting an optimal soft power of 10 (Figs. 5A and S1A). The eigenvalues between each module were then calculated for correlation analyses between modules (Fig. 5B) and between modules and traits. Highly connected genes within each module were determined by calculating the eigengene-based connectivity, kME (Fig. 5E). We performed a correlation analysis between the modules and single-cell sequencing data features. Single-cell data features were the activity scores (AUCell scores) of the transcription factors identified by SCENIC in each cell (Fig. 5C). Among them, the module with the strongest correlation with SREBF1 activity was epithelial cells-M1, with correlation coefficients of 0.91, 0.84, and 0.39 in the Normal, Primary cancer, and CRPC groups, respectively (Figs. 5C and S1B, C, D). The hub genes of Epithelial cells-M1 are shown in Fig. 5D. KEGG pathway enrichment analysis of the hub genes in Epithelial cells-M1 showed significant enrichment in ferroptosis and fatty acid metabolism-related pathways (Fig. 5F).

### Construction of a biochemical recurrence risk score for prostate cancer based on SREBF1 target genes combined with bulk RNA seq analysis

**Fig. 6:**
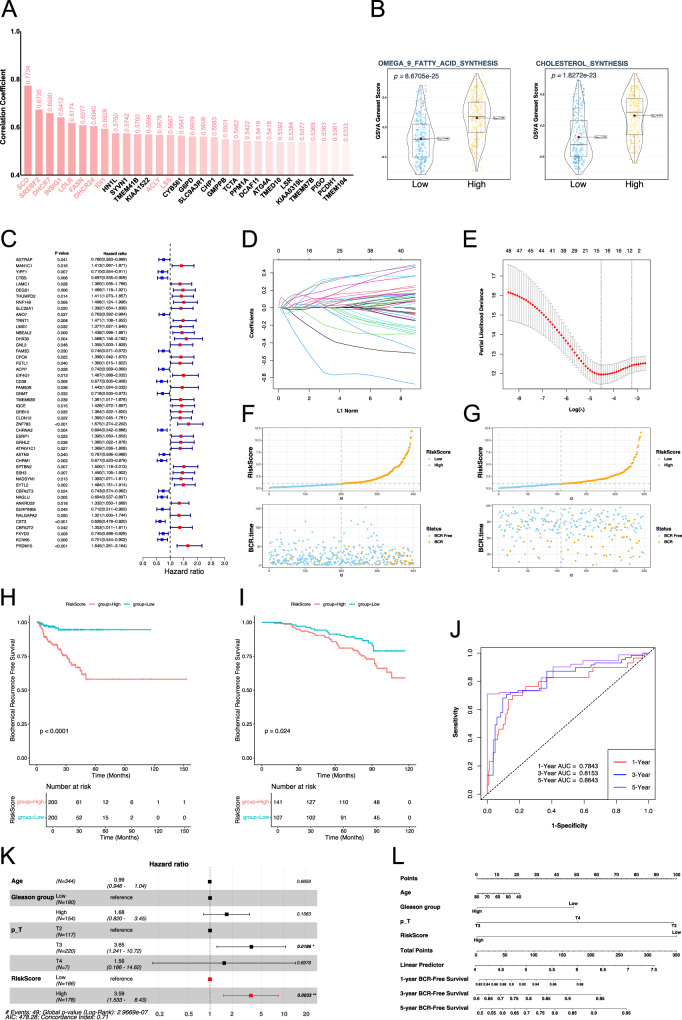
Bulk-RNA seq analysis based on SREBF1 and its target genes and risk scoring model associated with biochemical recurrence of prostate cancer. **A** Histogram of correlation analysis between all genes and SREBF1 expression in TCGA-PRAD. **B** Violin plot of gene sets GSVA scores. **C** Risk forest plot of genes associated with biochemical recurrence of prostate cancer obtained by univariate Cox regression analysis. **D** Coefficient path diagram of genes in Lasso regression analysis. **E** Cross-validation curve in Lasso regression analysis, nfolds = 10. **F** Distribution of risk scores and biochemical recurrence characteristics of samples in TCGA cohort. **G** Distribution of risk scores and biochemical recurrence characteristics of samples in GSE116918 cohort. **H** KM curve of biochemical recurrence based on risk score grouping in the TCGA cohort. **I** KM curve of biochemical recurrence based on risk score grouping in the GSE116918 cohort. **J** ROC curve of the risk score group-based prediction model for biochemical recurrence in the TCGA cohort. **K** Multivariate Cox regression analysis of risk scores and clinical data associated with biochemical recurrence. **L** Nomogram based on risk score and clinical data associated with biochemical recurrence. BCR biochemical recurrence, p_T pathological T stage, ROC Curve receiver operating characteristic curve, AUC area under curve, KM Curve Kaplan–Meier curve.

We performed a correlation analysis between all genes in the TCGA prostate cancer samples and SREBF1, and the results are shown in Fig. 6A. Among these, SCD1 showed the strongest correlation with SREBF1, with a correlation coefficient of 0.77. SCD-mediated MUFA synthesis is associated with anti-ferroptosis. Additionally, some genes directly related to cholesterol synthesis also have a high correlation, such as IDI1, LSS, and DHCR24. SREBF2 and SREBF1, both parts of the SREBPs family related to cholesterol metabolism, are often discussed together. ACAT2, HMGCS1, and HMGCR, key genes related to the MVK pathway in cholesterol synthesis, were highly correlated with SREBF1 (Fig. 6A and Table S3). Additionally, we used GSVA to analyze the activity of related gene sets in prostate cancer samples. Prostate cancer samples were divided into two groups based on the expression level of SREBF1. The GSVA score results showed that in the SREBF1 high expression group, the omega-9 fatty acid and the cholesterol synthesis pathways had higher activity (Fig. 6B).

Subsequently, we used the SREBF1 target gene to construct a risk score for the biochemical recurrence (BCR) of prostate cancer. First, a total of 48 genes associated with the biochemical recurrence of prostate cancer were screened using univariate Cox regression (Fig. 6C). Sixteen target genes associated with biochemical recurrence of prostate cancer were further screened out by Lasso regression, including MAN1C1, CTBS, LAMC1, DEGS1, TRNT1, DHX30, FSTL1, EIF4G1, FAM50B, GRB10, SPTBN2, NADSYN1, SYTL2, NAGLU, SERPINB5, and PRDM15 (Fig. 6D, E). In the univariate Cox analysis of these 16 genes associated with biochemical recurrence of prostate cancer, only three genes had a risk-reducing effect (Fig. S2). Subsequently, a multivariate Cox regression analysis based on these 16 genes was used to construct a risk score (Fig. 6F). The KM survival curve showed that the high-risk and low-risk groups, based on the median risk score of the TCGA cohort (0.9230274), had significant differences in BCR survival analysis, with the high-risk group having a higher risk of biochemical recurrence (Fig. 6H). We also validated the model using an external cohort (GSE116918), which showed that the high-risk group had a higher risk of biochemical recurrence (Fig. 6G, I). The ROC curve demonstrated the model efficiency of the risk score grouping based on TCGA cohort associated with BCR risk, with AUC values of 0.7843, 0.8153, and 0.8643 at one year, three years, and five years, respectively (Fig. 6J). We included clinical indicators, such as age, Gleason grouping (≤7 points for Gleason low-risk group), and pathological T stage, to compare their relationship with BCR risk with the risk grouping we constructed in multivariate Cox analysis. The T3 stage in pathological T stage was associated with increased BCR risk (HR = 3.65, *P* = 0.0186), and the high-risk group of the risk score was also associated with increased BCR risk (HR = 3.59, *P* = 0.0033) (Fig. 6K). Based on these features, a nomogram was constructed (Fig. 6L).

### The SREBF1 inhibitor Betulin significantly promotes ferroptosis in prostate cancer

Considering the regulation of metabolic reprogramming in prostate cancer by SREBF1 and its relationship with ferroptosis, we investigated the effect of the SREBF1 inhibitor Betulin on promoting ferroptosis in prostate cancer. RSL3, an inhibitor of the classical ferroptosis-resistant pathway, was used as a positive control to promote ferroptosis. Our experiments involved the androgen-sensitive prostate cancer cell line LNCaP and the castration-resistant prostate cancer cell line PC3.

**Fig. 7:**
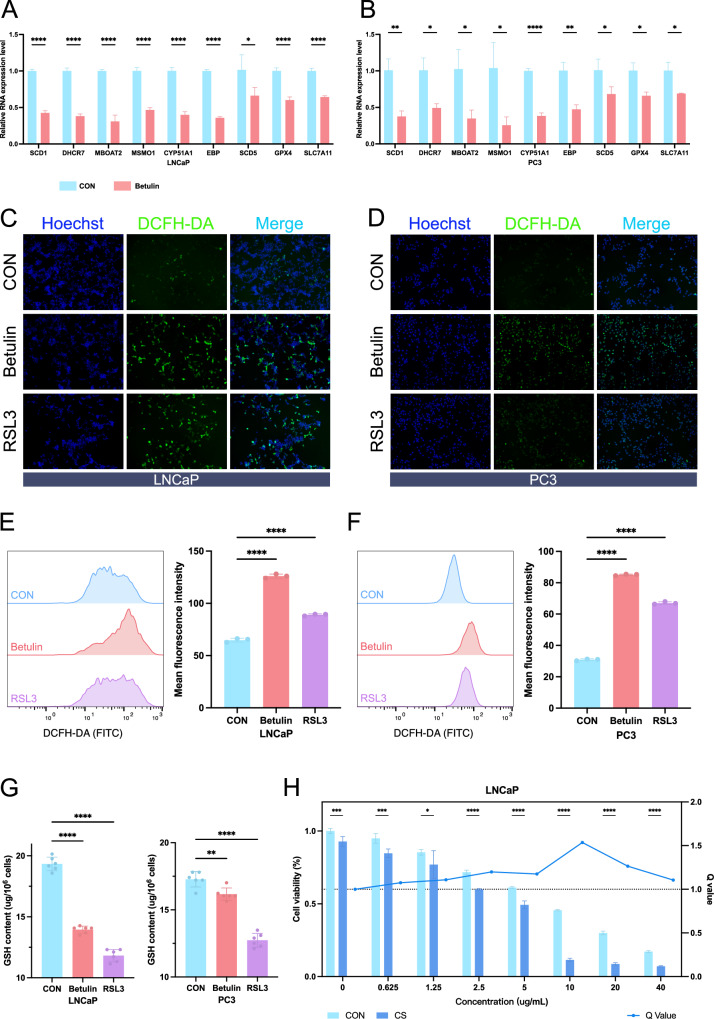
Validation of the SREBF1 inhibitor Betulin’s ability to promote ferroptosis in prostate cancer cells. qRT-PCR analysis of the expression of some SREBF1 target genes and ferroptosis-related genes in LNCaP (**A**) and PC3 (**B**) cell lines (*n* = 3). Fluorescence microscopy observation of ROS content in LNCaP (**C**) and PC3 (**D**) cells. Hoechst was used to stain the nuclei, and ROS was visualized using the DCFH-DA probe (*n* = 3). Cell flow cytometry analysis of ROS content in LNCaP (**E**) and PC3 (**F**) cells. Quantitative comparison by Mean Fluorescence Intensity (MFI) (*n* = 3). **G** Measurement of GSH content in LNCaP and PC3 cells (*n* = 6). **H** Cell viability assay in LNCaP cells after androgen deprivation culture and Betulin treatment. The left y-axis represents cell viability, and the right *y*-axis represents the *Q* value calculated for each concentration group (*n* = 3). CS charcoal Stripped. Data were presented as mean ± SD. (ns, *P* ≥ 0.05; *P < 0.05; **P < 0.01; ***P < 0.001; ****P < 0.0001).

We verified the expression of SREBF1 target genes and ferroptosis-related genes in drug-treated cell lines using quantitative real-time polymerase chain reaction (qRT-PCR). In the LNCaP cell line, the expression of target genes such as SCD1, DHCR7, MSMO1, CYP51A1, and EBP decreased significantly, and GPX4 decreased to a certain extent (Fig. 7A). However, in the PC3 cell line, ferroptosis-related genes such as SCD5, GPX4, and SLC7A11 were not affected much in the Betulin group (Fig. 7B). Next, we detected intracellular reactive oxygen species (ROS) to determine the degree of ferroptosis in prostate cancer cells after Betulin treatment. Fluorescence microscopy revealed a significant increase in ROS levels in both LNCaP and PC3 cells following Betulin treatment (Fig. 7C, D). The results of intracellular ROS detection using flow cytometry were consistent. In LNCaP cells, the Mean Fluorescence Intensity (MFI) of ROS detection in the Betulin group was significantly increased (126), compared with 89 in the RSL3 group and 64.73 in the control group (Fig. 7E). Similarly, in the PC3 cell line, the average MFI of ROS detection in the Betulin and RSL3 group were 85.07 and 67, respectively, which were significantly higher than the 30.87 in the control group (Fig. 7F). Intracellular glutathione (GSH) levels were measured to observe intracellular oxidative stress. In LNCaP cells, GSH decreased significantly in the Betulin group and RSL3 group, with levels of 13.94 μg/10^6^ cells and 11.82 μg/10^6^ cells, respectively, compared to 19.33 μg/10^6^ cells in the control group. Similarly, in PC3 cells, GSH also decreased in the Betulin group and RSL3 group, with levels of 16.16 μg/10^6^ cells and 12.73 μg/10^6^ cells, respectively, compared to 17.28 μg/10^6^ cells in the control group (Fig. 7G). Finally, we examined whether castration and Betulin treatment had a synergistic effect on hormone-sensitive prostate cancer cell lines. We observed that the Q value was greater than 1 at Betulin concentrations ranging from 0.625 μg/mL to 40 μg/mL, indicating a synergistic effect between the two treatments. The Q value was highest around 10 μg/mL, indicating the most significant synergistic effect (Fig. 7H).

### Betulin decreased mitochondrial membrane potential in prostate cancer cells and exhibited synergistic therapeutic effects with docetaxel

**Fig. 8:**
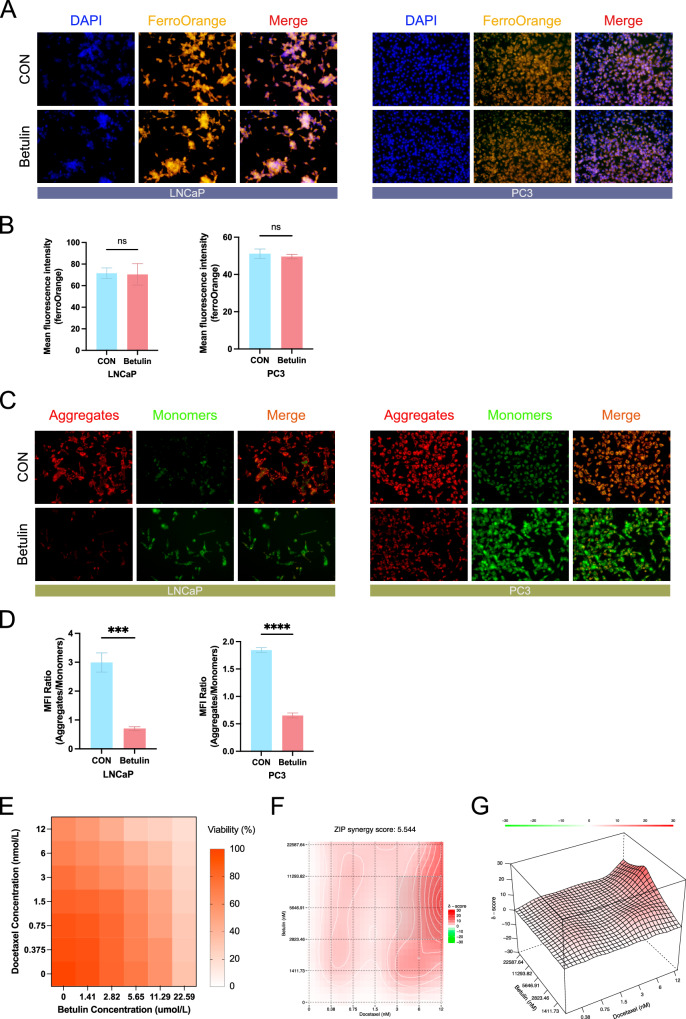
Changes in ferroptosis-related characteristics of prostate cancer cells treated with Betulin and the synergistic therapeutic effect of Betulin and docetaxel. **A** Intracellular ferrous ion staining was performed using the ferrous ion probe FerroOrange, and cell nuclei were stained using DAPI (*n* = 3). **B** Quantification of ferrous iron staining. **C** Intracellular mitochondrial membrane potential staining, using JC-1 staining, aggregates are in red, and monomers are in green (*n* = 3). **D** Quantification of JC-1 staining, results were shown as the ratio of aggregates to monomers. **E** Heat map of cell activity, with the horizontal axis representing the betulin treatment concentration and the vertical axis representing the docetaxel treatment concentration (*n* = 5). **F** Heat map of drug combination synergistic effects, presented as the analysis results of the zero interaction potency (ZIP) model, completed by SynergyFinder. **G** 3D heat map of drug combination synergy, presented as the analysis result of zero interaction potency (ZIP) model, completed by SynergyFinder. ZIP zero interaction potency (ZIP). Data were presented as mean ± SD. (ns, *P* ≥ 0.05; *P < 0.05; **P < 0.01; ***P < 0.001; ****P < 0.0001).

Previous research has shown that the SREBF1 inhibitor Betulin can significantly increase ROS levels and decrease GSH levels in prostate cancer cells. Detection of mRNA levels revealed a decrease in the expression of genes closely related to lipid metabolism and ferroptosis resistance, including SCD1, DHCR7, and MSMO1. Since ferroptosis is closely related to the intracellular ferrous ion content, we examined whether Betulin affects intracellular ferrous ion levels using intracellular ferrous ion staining. The results indicated that the intracellular ferrous ion content did not change significantly in either the PC3 or LNCaP cell lines (Fig. 8A, B), suggesting that Betulin-mediated ferroptosis is not related to cellular ferrous ion content. Subsequently, we observed changes in the mitochondrial membrane potential, which decreased in both PC3 and LNCaP prostate cancer cell lines (Fig. 8C, D). Currently, ADT is the primary treatment for prostate cancer; however, almost all patients develop castration resistance after a period of treatment, making chemotherapy drugs crucial for advanced prostate cancer. Finally, we investigated the potential synergistic effects of Betulin and docetaxel, which are chemotherapeutic drugs commonly used in advanced prostate cancer. Different concentrations of Betulin and docetaxel were combined to treat PC3 cell lines (Fig. 8E). Synergy analysis revealed a synergistic therapeutic effect, with a ZIP synergy score of 5.54 (Fig. 8F, G).

### Verification of Betulin’s therapeutic effect and its chemosensitizing effect on docetaxel in vivo

**Fig. 9:**
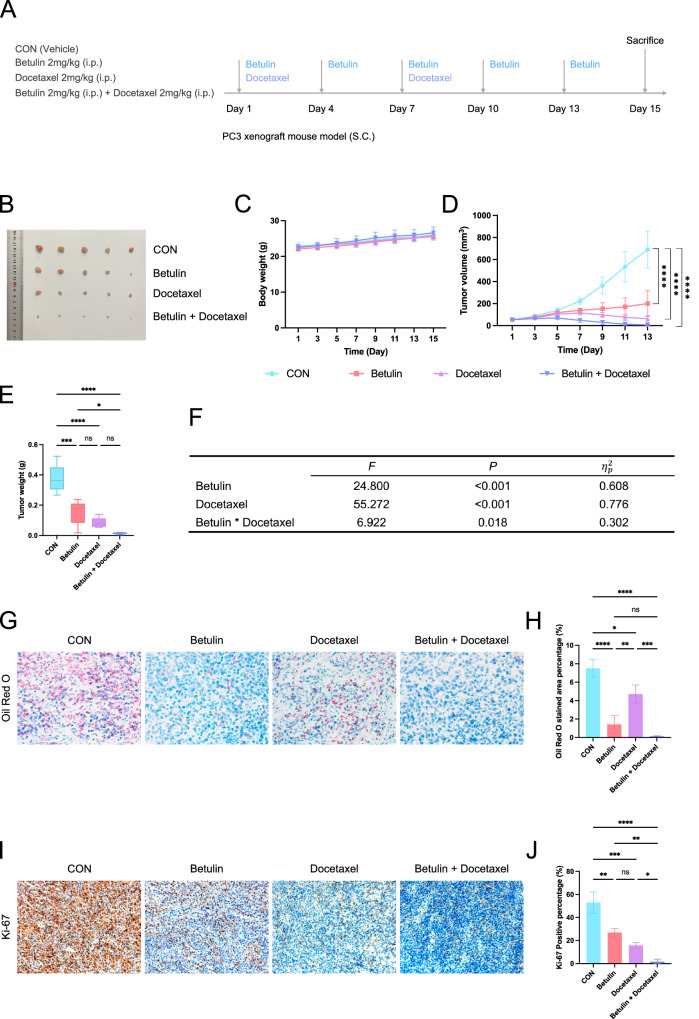
In vivo experiment of betulin combined with docetaxel in the treatment of PC3 prostate cancer subcutaneous xenograft tumor model. **A** Schematic of experimental design and procedure (*n* = 5). **B** Image of the tumor after treatment. **C** Body weight change curve of mice during the treatment process. **D** Tumor size change curve during treatment. **E** Tumor weight after treatment completion. **F** Results of the variance analysis of the factorial design for treatment effects. **G** Oil red O staining of tumor tissue after treatment. **H** Quantification of Oil Red O staining. **I** Ki-67 staining of tumor tissue after treatment. **J** Quantification of Ki-67 staining. i.p. intraperitoneal Injections, SC subcutaneous. Data were presented as mean ± SD. (ns, *P* ≥ 0.05; *P < 0.05; **P < 0.01; ***P < 0.001; ****P < 0.0001).

We constructed a PC3 prostate cancer subcutaneous xenograft tumor model and used Betulin, docetaxel alone, or a combination of Betulin and docetaxel to observe their therapeutic effects (Fig. 9A). Betulin and docetaxel moderately inhibited tumor growth when used alone, whereas the tumor growth inhibitory effect was more pronounced when used in combination (Fig. 9B, D, E). No significant effect was noted on the body weight of the mice, whether they were used alone or in combination (Fig. 9C). The analysis of variance results of the factorial design showed that Betulin treatment (*F* = 24.800, *P* < 0.001, partial eta squared = 0.608) and docetaxel treatment (*F* = 55.272, *P* < 0.001, partial eta squared = 0.776) had significant main effects. Notably, the interaction effect of Betulin and docetaxel treatment was significant (*F* = 6.922, *P* = 0.018, eta squared = 0.302) (Fig. 9F). Oil Red O staining showed that Betulin treatment significantly reduced the level of tissue lipid droplets (Fig. 9G, H), and Ki-67 staining indicated that Betulin and docetaxel treatment alone inhibited tumor proliferation, with the combination of Betulin and docetaxel showing a stronger inhibitory effect (Fig. 9I, J).

## Discussion

Metabolic reprogramming is a hallmark of cancer that encompasses alterations in glucose, lipid, and amino acid metabolism [19]. Cancer cells adapt their energy metabolism to fulfill their high energy demands, often altering their metabolic pathways to suit their needs. Warburg first discovered that cancer cells prefer glycolysis for their energy supply, a phenomenon known as the “Warburg effect” [20]. Despite being less efficient in ATP production, glycolysis offers several advantages to tumor cells, marking the earliest chapter in the understanding of tumor metabolic changes.

Metabolic reprogramming in cancer affects various biological behaviors of cancer cells, promoting proliferation, metastasis, and drug resistance. The reprogramming of lipid metabolism is particularly important in cancers [21, 22]. Lipids, including fatty acids and cholesterol, play critical roles in energy storage, metabolism, and various biological functions within cells, such as serving as the main components of cell membranes and acting as signaling molecules [23]. In prostate cancer, the unique metabolic features of high zinc concentrations and citrate accumulation and production further emphasize the importance of lipid metabolism reprogramming. However, the vulnerability of prostate cancer to metabolic changes presents new therapeutic opportunities and directions for cancer treatment.

Ferroptosis is an iron-dependent form of cell death characterized by lipid peroxidation and is closely linked to lipid metabolism [24]. Resistance to ferroptosis is a common feature of tumor cells. Elevated levels of ROS associated with ferroptosis resistance can induce genetic mutations and enhance the malignancy of tumor cells. Additionally, tumor cells can significantly bolster their defence against oxidative stress through ferroptosis resistance, enabling their survival and resistance to drug treatments [25].

In this study, we investigated the relationship between metabolic reprogramming and ferroptosis in prostate cancer regulated by SREBF1. First, we analyzed the characteristics of different cell types in prostate cancer samples using single-cell sequencing and identified high levels of activation of pathways such as the PI3K-AKT pathway, P53 pathway, MYC pathways, androgen response, glycolysis, fatty acid metabolism, and bile acid metabolism in prostate cancer epithelial cells. Of particular interest were lipid-related metabolic pathways such as androgen response, glycolysis, fatty acid metabolism, and bile acid metabolism. Next, we observed characteristic differences in epithelial cells among Normal, Primary cancer, and CRPC samples and found that epithelial cells derived from primary cancer samples mainly exhibited higher activity in the androgen response, fatty acid metabolism, cholesterol metabolism, and other pathways. Additionally, epithelial cells derived from both primary cancer and CRPC samples showed higher activity in glycolysis and the MTORC1 pathway.

We observed a significant upregulation of ACLY, FASN, and SCD1 in primary cancer and CRPC samples. ACLY is the first enzyme in the citric acid pathway, which leads to lipid metabolism. The significantly upregulated expression of ACLY in epithelial cells from primary cancer and CRPC samples suggests increased utilization of citric acid in the lipid synthesis pathway during tumorigenesis. Furthermore, FASN and SCD1, both associated with fatty acid metabolism, were significantly upregulated in primary cancer and CRPC samples. FASN is a key enzyme that mediates fatty acid synthesis, whereas SCD1 plays a crucial role in monounsaturated fatty acid (MUFA) synthesis. SCD1, a key regulator of the fatty acid metabolic pathway, regulates ferroptosis resistance [26].

We further investigated the relationship between transcription factors and metabolic changes by analyzing the transcription factor regulatory network. Among the 30 transcription factors with high activity in the epithelial cells of single-cell samples, SREBF1 caught our attention.

SREBPs are a family of basic helix-loop-helix leucine zipper transcription factors that regulate de novo synthesis of fatty acids and cholesterol, as well as cholesterol uptake, making them closely associated with lipid metabolism [27–29]. Our analysis revealed that the target genes of SREBF1 included HMGCS1, DHCR7, SC5D, SCD1, ACLY, FASN, and LDLR.

We observed that SREBF1 activity was highest in epithelial cells from primary cancer samples and accordingly divided cells into SREBF1_POS and SREBF1_NEG groups based on the transcriptional activity of epithelial cells to observe the differences between them. Significant differences were evident in several fatty acid- and cholesterol-related pathways that also play crucial roles in ferroptosis resistance. One of the most significant differences was observed in the Cholesterol Metabolism pathway, specifically in the steps from Lanosterol to cholesterol synthesis. The exact reaction sequence between lanosterol and cholesterol is not yet fully understood. Based on the reaction sequence of DHCR24, the synthesis pathway from lanosterol to cholesterol can be divided into two pathways: the Bloch and the Kandutsch–Russell pathways [30]. The upregulation of cholesterol synthesis is conducive to the synthesis of endogenous androgens, which activate AR independently of exogenous androgens, thereby promoting the proliferation of prostate cancer. AR activation may further upregulate the activity of SREBF1, forming a positive feedback loop [31, 32].

Furthermore, Omega9 Fatty Acid Synthesis, which produces MUFA, has been shown to confer ferroptosis resistance. Interestingly, there were significant differences in pathways related to the Mevalonate (MVA) pathway, including the Mevalonate Arm of Cholesterol Biosynthesis Pathway and the Mevalonate Pathway. The MVA pathway is the first stage in cholesterol synthesis, generating the structural precursors of the steroid substances, isopentenyl pyrophosphate (IPP), and dimethylallyl pyrophosphate (DMAPP) from acetyl-CoA.

The MVA pathway is an important contributor to selenoprotein synthesis, and GPX4 is a selenoprotein with selenocysteine in its active center. Because the genetic code for selenocysteine is UGA, which is the same as the stop codon, a specific transporter is required to insert selenocysteine into GPX4 [33]. This transporter is a selenocysteine tRNA that contains isopentenyladenosine and can decode the genetic code for selenocysteine by precisely inserting selenocysteine into the corresponding protein. However, the maturation of selenocysteine tRNA requires tRNA-isopentenyl transferase to catalyze the transfer of the isopentenyl group from isopentenyl pyrophosphate (IPP) to specific adenine sites on selenocysteine tRNA precursors [34]. Therefore, upregulation of this pathway can promote resistance to ferroptosis. We also observed that the cholesterol synthesis pathways, including the MVA pathway, were highly activated in the SREBF1_POS group.

In the combined Bulk-RNA seq analysis, we observed that genes closely related to SREBF1 were mainly involved in pathways related to fatty acid synthesis and cholesterol synthesis, with some of these genes acting as key regulators in these pathways. For instance, SCD1, which showed the highest correlation with SREBF1, is crucial for regulating MUFA synthesis, whereas ACLY and FASN are key genes involved in fatty acid synthesis. These findings were consistent with those of our single-cell sequencing analyses. Survival analysis further indicated that high expression of SCD1, ACLY, DHCR7, and SREBF1 was associated with poor prognosis.

Interestingly, studies by Li et al. and Freitas et al. showed that the cholesterol synthesis precursor 7-dehydrocholesterol (7-DHC) can confer resistance to ferroptosis [35, 36]. The average expression levels of the genes involved in 7-DHC synthesis, including EBP and SC5D, were higher than those in DHCR7. Li et al. suggested that the high expression of DHCR7 promotes ferroptosis. However, we believe that this should be discussed in this context. Firstly, when the activity of genes involved in 7-DHC synthesis is significantly higher than that of DHCR7, excess of 7-DHC is available to resist ferroptosis. Secondly, the continuous and strong activation of the cholesterol synthesis pathway leads to sufficient 7-DHC production, regardless of the level of DHCR7 expression level. Moreover, cholesterol synthesis in prostate cancer is conducive to the production of endogenous steroid hormones, thereby promoting tumor progression.

It is noteworthy that our results also revealed significant activity and enrichment of SREBF2. Furthermore, correlation analysis demonstrated a strong association between the expression levels of SREBF2 and SREBF1, suggesting that these two factors may interact rather than function independently. SREBF1 primarily participates in the synthesis of fatty acids and cholesterol as well as cholesterol uptake, while SREBF2 predominantly regulates cholesterol metabolism and absorption [22]. Elevated activation of cholesterol synthesis pathways driven by SREBPs, including the mevalonate pathway, not only enhances ferroptosis resistance in prostate cancer but also increases cholesterol flux towards steroid hormone synthesis [37]. Steroid hormones, such as testosterone and dihydrotestosterone (DHT), further contribute to the progression of prostate cancer.

A biochemical recurrence risk scoring model based on the SREBF1 target gene identified several genes closely related to the biochemical recurrence of prostate cancer and demonstrated a good predictive effect. However, the mechanisms underlying the biochemical recurrence of prostate cancer require further investigation.

Finally, we investigated the effect of the SREBF1 inhibitor Betulin on promoting ferroptosis in prostate cancer. Previous studies have confirmed that Betulin exhibits significant anti-prostate cancer activity [38]. Here, we explored its effect on ferroptosis in tumor cells. Our results demonstrated that treatment with Betulin led to a significant down-regulation of SREBF1 target genes associated with ferroptosis resistance at the genetic level. Additionally, intracellular ROS levels increased significantly, whereas GSH content decreased. Furthermore, the combination of castration treatment and Betulin treatment exhibited a synergistic therapeutic effect. In addition, our experiments showed no significant changes in the intracellular ferrous ion content, whereas the mitochondrial membrane potential decreased significantly after Betulin treatment. Ferrous ions play a critical role in ferroptosis [10]. Notably, this study observed no changes in intracellular ferrous ion levels following the application of the SREBF1 inhibitor. This finding suggests that ferroptosis resulting from SREBF1 inhibition is not independent of the regulation of ferrous ion levels. Betulin and docetaxel exert synergistic therapeutic effects. In vivo experiments verified the excellent therapeutic effect of Betulin and its chemosensitizing effect on docetaxel, suggesting that SREBF1 inhibitors may have a promising therapeutic potential in prostate cancer.

Some limitations of this study should be noted. Although the Harmony method was used to correct for the batch effect, the batch effect between different datasets cannot be ignored. Secondly, scRNA-seq studies with more patients and more cell numbers are conducive to eliminating individual differences between patients. Therefore, other mechanisms of ferroptosis resistance based on SREBPs still need to be explored and biologically verified. Our study provided directions and potential therapeutic targets for prostate cancer for future research.

In conclusion, our study revealed the role of SREBF1-mediated metabolic reprogramming in prostate cancer and its association with ferroptosis resistance. By combining single-cell sequencing and Bulk-RNA analysis, we demonstrated that the metabolic changes regulated by SREBF1 promote ferroptosis resistance in prostate cancer. Moreover, we found that SREBF1 inhibitors have excellent chemosensitizing effects in prostate cancer therapy, highlighting their potential for therapeutic applications in prostate cancer.

## Materials and methods

### Data acquisition and processing

Prostate cancer single-cell RNA sequencing data were obtained from the GEO datasets GSE193337 and GSE137829. GSE193337 includes normal prostate tissue, prostate cancer RP tissue, and GSE137829 includes CRPC-derived tumor tissue. Bulk RNA sequencing data were obtained from the TCGA database. Data analysis and processing were conducted using the R language (version 4.2.1) and Python. The Seurat package (v5.0.3) was utilized for single-cell sequencing analysis and visualization, while PySCENIC was used for the prediction of transcriptional regulatory factors from single-cell sequencing data.

### Single-cell RNA sequencing analysis

We initially merged single-cell sequencing data from the GSE193337 and GSE137829 datasets. Quality control was performed on the merged data using the following criteria: (1) Cells with fewer than 200 measured genes (min.features = 200) and genes covered by fewer than 3 cells (min.cells = 3) were filtered out. (2) Cells with gene expression counts below 201 (considered low-quality cells) or more than 8000 genes (indicating potential doublets) were excluded. (3) Cells with more than 20% of unique molecular identifiers (UMIs) derived from the mitochondrial genome were also excluded. (4) Mitochondrial genes, ribosomal genes, hemoglobin genes, and MALAT1 were removed. The Harmony package was used to remove batch effects between single-cell datasets from different sources. Data dimensionality reduction was performed using the RunPCA method, and 25 principal components were used for subsequent analyses. Nonlinear dimensionality reduction and result presentation were achieved using UMAP and tSNE. Cell clusters were manually annotated based on knowledge and relevant literature.

### Single-cell RNA sequencing transcription factor analysis

We employed PySCENIC to analyze and identify transcriptional regulators based on single-cell sequencing data [39]. The analysis and result visualization were performed in R using the SCENIC package. The GRNboost algorithm was used to construct a co-expression network between transcription factors and candidate target genes. TF-motif enrichment analysis was performed to identify direct targets of the transcription factors. Each processed TF and its potential direct target genes were used as regulons for subsequent analyses. AUCell was used to score all genes in each regulon. The resulting score is the Area Under Curve (AUC), which represents the “activity” of regulons in each cell.

### hdWGCNA

hdWGCNA (v0.3.03) analysis was performed on all epithelial cell subpopulations. The optimal SoftPower was determined using the TestSoftPowers() function. Correlation analysis between modules and features was conducted using the ModuleTraitCorrelation() function. Transcription factors were identified and screened by SCENIC. The analysis involved adding the transcription factor AUCell activity scores to the metadata in the Seurat object, with module features labeled as “hMEs”. Finally, KEGG pathway enrichment analysis was performed on the Hubgenes of specific module.

### Bulk-RNA Seq analysis

RNA-seq data and clinical data were obtained from the XENA and TCGA databases. Gene set expression activity scores in different samples were analyzed using GSVA. To construct the risk score, the target genes of SREBF1 identified by SCENIC were first analyzed using univariate Cox regression analysis to identify genes significant with biochemical recurrence of prostate cancer. LASSO regression was then used to further filter these genes for risk score construction. The coefficients used to calculate the risk score were derived from the multivariate Cox analysis. External validation was performed using the GEO dataset (GSE116918). Risk grouping was based on the median risk score calculated from the TCGA cohort, which distinguished between high-risk and low-risk groups. A nomogram was constructed by performing multivariate Cox regression analysis on both the risk grouping and clinical data. Samples with missing data were excluded from the cohort.

### Cell culture and drug treatment

Cell lines were cultured in a humidified incubator at 37 °C with 5% CO_2_. They were obtained from Procell Life Science & Technology Co., Ltd and authenticated by STR cell sequencing to confirm their identity and ensure that they were free of mycoplasma contamination. RPMI-1640 medium (Procell, PM150110) supplemented with 10% fetal calf serum (Procell, 164210-50) was used for cell culture, and charcoal-stripped serum was used to simulate androgen-deprived conditions. Betulin was added to the culture medium at a concentration of 5 μg/mL [38]. RSL3 was used as the positive control for ferroptosis induction at a concentration of 0.1 μM.

### Quantitative real-time polymerase chain reaction (qRT-PCR)

RNA was extracted using TRIzol reagent (Invitrogen). Subsequently, cDNA was synthesized following the manufacturer’s protocol (Evo M-MLV RT Mix Kit with gDNA Clean for qPCR Ver.2, AG11728). Real-time PCR was performed using the Applied Biosystems 7900 Real-Time PCR System (Thermo Scientific) and SYBR Green PCR Master Mix (Roche). β-actin was used as the internal control, and the primer sequences are listed in Table S4.

### Detection of intracellular ROS

The ROS fluorescent probe 2′,7′-Dichlorodihydrofluorescein diacetate (DCFH-DA) (Aladdin, H131224) was used for intracellular ROS detection. DCFH-DA was added to the cells to achieve a final concentration of 20 μM. During cell flow cytometry analysis, the cells were incubated with DCFH-DA for 30 min, and detection was performed at an excitation wavelength of 488 nm and an emission wavelength of 525 nm. For fluorescence microscopy, the cells were incubated with DCFH-DA for 30 min, and the nuclei were stained with Hoechst stain.

### Detection of intracellular glutathione

After experimental treatment, the cells were collected, counted, and subjected to GSH detection. GSH reacts with 5,5’-dithiobis-2-nitrobenoic acid (DTNB) to produce GSSG, which was detected at a wavelength of 412 nm. The detection procedure was performed according to the manufacturer’s instructions (Solarbio, BC1170).

### Cell counting kit-8 (CCK-8) and drug synergistic analysis

The CCK-8 test kit was purchased from ApexBio Technology (K1018). This procedure was carried out according to the manufacturer’s instructions. To explore the effects of Betulin and androgen deprivation on cells, the charcoal-adsorbed serum group was used as the androgen deprivation group, and Betulin medication group was set with a concentration gradient of 0, 0.625, 1.25, 2.5, 5, 10, 20, and 40 µg/mL. The test was performed after 48 hours of culture. The Jin Zhengjun method was used to determine whether there is a synergistic inhibition of cell proliferation between androgen-deprived culture and Betulin treatment [40–42]. The specific formula: *Q* = *E*_(A+B)_/(*E*_A_ + *E*_B_–*E*_A_·*E*_B_), *E*_A_ is the effect of A treatment, *E*_B_ is the effect of B treatment, and *E*_(A+B)_ is the joint treatment effect. A Q value greater than 1 indicates a synergistic effect. When investigating the effects of Betulin and docetaxel on PC3 cells, a Betulin treatment group (with a concentration gradient of 0.625, 1.25, 2.5, 5, and 10 µg/mL), a docetaxel treatment group (with a concentration gradient of 0.375, 0.75, 1.5, 3, 6, and 12 nmol/L), and a combined treatment group were set up. The concentration of Betulin was converted to a molar concentration. SynergyFinder was used to analyze the synergistic effects of the two treatments when used in combination [43].

### Ferrous ions and mitochondrial membrane potential staining

FerroOrange was used as a fluorescent probe for ferrous ions to perform fluorescence imaging of ferrous ions in cells. DoJinDo (F374) was used as the product, and the procedure was performed according to the manufacturer’s instructions. JC-1 was used as a fluorescent probe to detect changes in the cell membrane potential. Beyotime (C2006) was used for detection according to the manufacturer’s instructions.

### Animal experiment

All mice were housed under specific pathogen-free (SPF) conditions. Mice had ad libitum access to water and standard rodent chow. To establish a PC3 prostate cancer subcutaneous tumor mouse model, the PC3 cell line was subcutaneously inoculated into the dorsal flank of 5–6 week-old male nude mice, with 3 × 10^6^ cells inoculated per mouse. The tumor size (including length and width) was measured daily, and the tumor volume was estimated using the formula *V* = (width^2^ × length)/2. When solid tumors became palpable, the mice were randomly divided into four groups: (1) Control group (vehicle group); (2) Betulin group: intraperitoneal injection of 2 mg/kg Betulin once every three days; (3) Docetaxel group: intraperitoneal injection of 2 mg/kg docetaxel once a week; (4) Betulin and Docetaxel combination group: the dose and frequency of administration were the same as those of the single drug groups. The operations, and vehicles of the injected drugs were consistent across all four groups. After 14 days of treatment, the mice were sacrificed, and the tumors were dissected for subsequent experiments. Experimenters were blinded to treatment allocation. Data collection and analysis were performed on anonymized datasets to ensure objectivity. For Oil Red O and Ki-67 immunohistochemical staining, fresh tumor tissues were dehydrated, and frozen sections were prepared. An Oil Red O stock solution was prepared by dissolving 0.3 g of Oil Red O powder in 50 ml of isopropanol. The Oil Red O stock solution was then mixed with distilled water at a ratio of 3:2 to obtain the Oil Red O working solution. Before use, the working solution was filtered with a 0.22-micron microporous membrane. The sections were rinsed with 60% isopropanol, stained with the Oil Red O working solution for 15 min, washed with 60% isopropanol until the background is colorless, and rinsed with distilled water to remove the excess stain. Finally, hematoxylin was used to stain the nucleus.

### Graphing and statistical analysis

Graphing and statistical analyses were primarily conducted using R language, RStudio, SPSS, and Prism 9. The figures were formatted using Affinity Designer and Affinity Photo. Fluorescence, immunohistochemistry, and Oil Red O staining were quantified using Image J and Fiji software. All experiments were performed independently at least three times, and the number of independent experiments is reported in the figure legend. Statistical analyses included unpaired two-tailed *t*-tests to compare differences between two groups and one-way ANOVA to compare differences between multiple groups. Unless otherwise stated, *P* < 0.05 was considered statistically significant. No statistical methods were used to predetermine sample size.

## Supplementary information

Supplementary Figure S1

Supplementary Figure S2

Supplementary Table S1

Supplementary Table S2

Supplementary Table S3

Supplementary Table S4

## Data Availability

All data are mentioned in the methods.
