# Supplementary figures and images for: SREBF1-based metabolic reprogramming in prostate cancer promotes tumor ferroptosis resistance
Source: Cell Death Discov. 2025 Feb 23;11:75. doi: 10.1038/s41420-025-02354-7 (PMC11847930; doi:10.1038/s41420-025-02354-7)

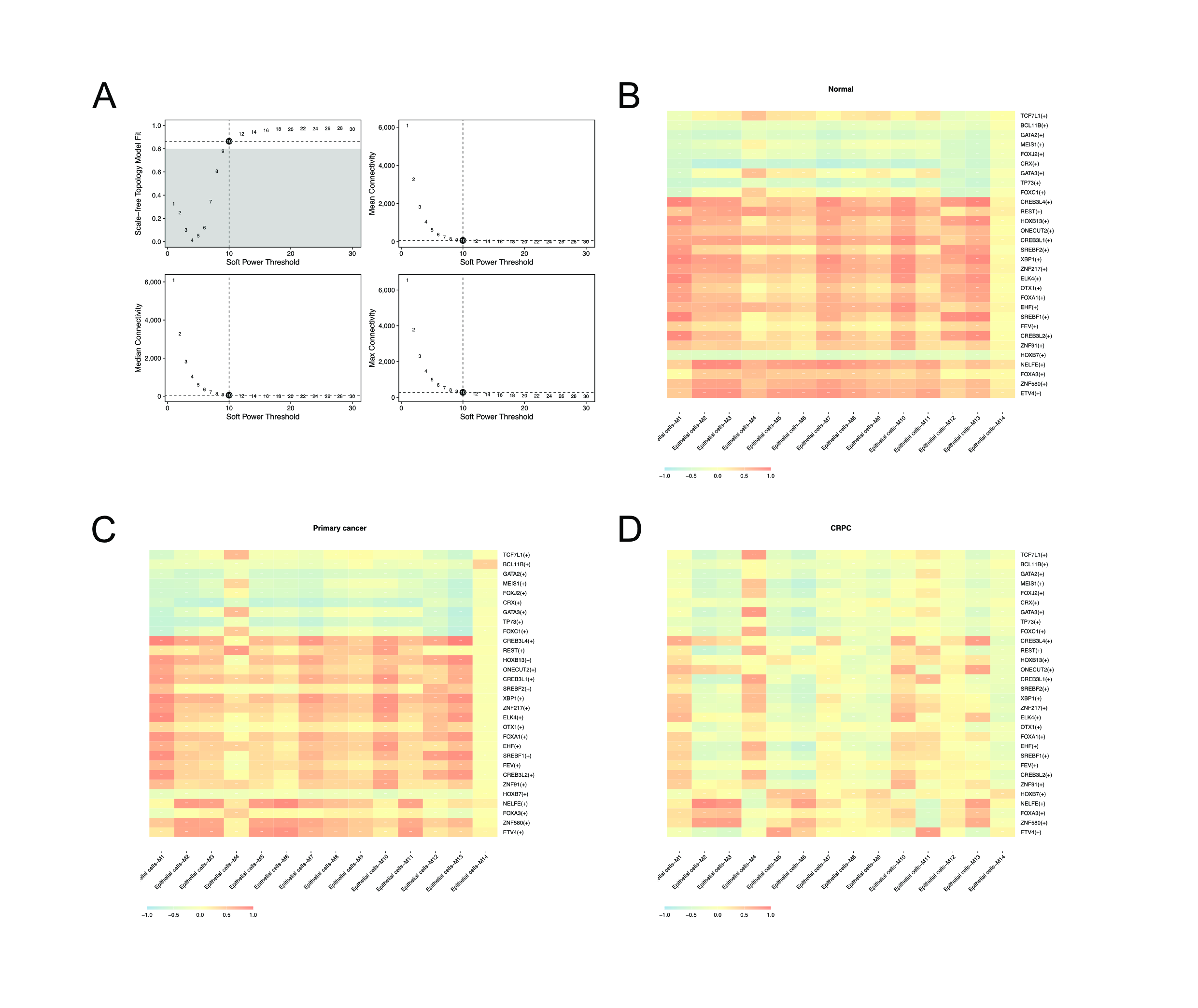

Supplement: Supplementary file 1 — Supplementary Figure S1 [file 41420_2025_2354_MOESM1_ESM.tif]

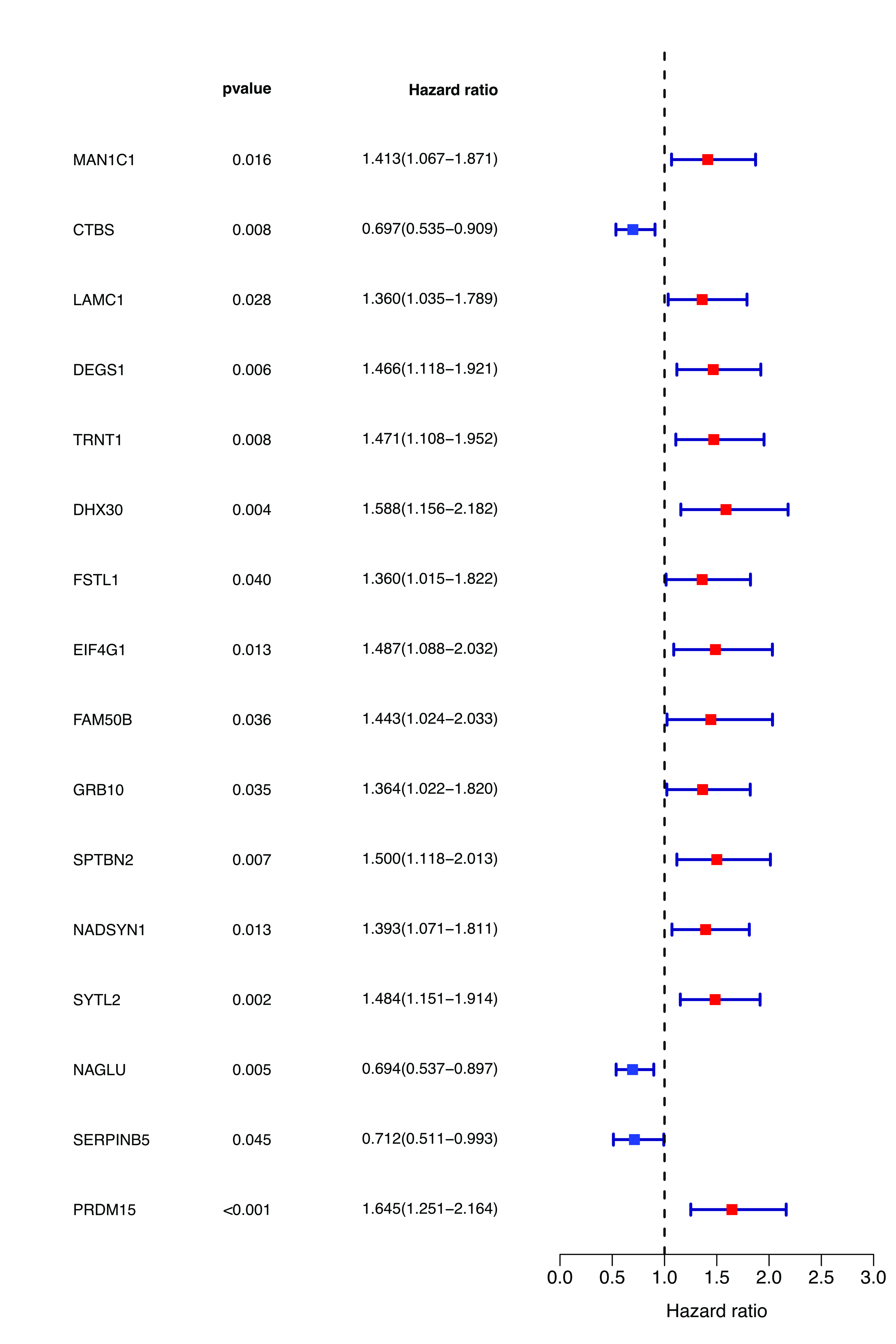

Supplement: Supplementary file 2 — Supplementary Figure S2 [file 41420_2025_2354_MOESM2_ESM.tif]
